# London Forceps

**Published:** 1845-12

**Authors:** 


*London Forceps.*—Engaged in the same pursuit, and united by the ties of a common brotherhood, we cannot regard with indifference—although living under a different form of government, and separated from them by the broad Atlantic, the doings of our European brethren. We hail with the most lively joy, every effort made by them for the advancement of that branch of the curative art, in the practice and cultivation of which, we are mutually engaged, and the failure of any enterprise undertaken there, calculated to promote this praiseworthy end, is as deeply and sincerely regretted by us, as it is by them. Therefore, it is with feelings of the most unfeigned regret, that we announce, that the publication of that ably conducted periodical—the *London Forceps*, has ceased. Although the experiment of the publication of a journal devoted to the interests of the profession in England, had been previously made, and failed, we had hoped, that the Forceps, phœnix-like, rising up from the ashes of the British Quarterly, would have become the rallying point of our transatlantic brethren—that devoted as it was to the advancement of the science and art of dental surgery, and the elevation of the standard of professional qualification, they would have sustained it. In the city of London alone, there are dentists enough, if we are correctly informed as to the number, to have done this.

But composed as the dental profession at present is, all over the world, of men, many of whom, have been brought up to occupations requiring but little or no education, and are wholly unaccustomed to reading, it is hardly to be expected that such of its members should feel the necessity of extending the range of their knowledge. Hence they regard the expenditure of money for books or scientific periodicals, as worse than useless; and we are inclined to believe, that those who entertain this opinion are right. It is exceedingly questionable whether they would be profited by books, and it may be, that they act wisely in refusing to encumber themselves with such useless rubbish, inasmuch as reading upon the subject, would only expose their ignorance, and thus lower them in their own estimation. We are happy to know, however, that the ranks of the profession are rapidly filling up with a different class of individuals—with men of intelligence and education, who are enriching the art by scientific researches and enlightened experience. Although the number of this latter class is, comparatively, small, yet they are rapidly changing the character of the pursuit, and securing for it a consideration which it has not heretofore enjoyed. As this description of practitioners become more numerous, will dental periodical literature be more liberally patronized, for, it is the ambition of the scientific dentist, to avail himself of every means of information within his reach. He will not be contented with the reading of one, nor half a dozen works upon the subject; he will have every thing that is written upon it, if within his ability, that he may be able to give his patients the benefit of the discoveries and improvements in practice that are daily being made. It was said, we believe, by the celebrated Dr. Rush, that "when a physician quits reading, he should quit practice," and the remark will apply with as much force to the dental as to the medical practitioner.

With regard to the importance of a medium of intercommunication between the members of the dental profession, through which such discoveries and improvements may be rendered available to all, we should not think there could be two opinions. It is too apparent to admit of doubt, and yet, how little interest, do the majority of dentists manifest for such vehicles of knowledge? In the United States, not more than one out of six manifests any at all. We have had an opportunity of ascertaining this fact, and therefore speak advisedly upon the subject.

Having been connected with the publication of this Journal during the entire period of its existence, we cannot be supposed wholly ignorant of the difficulties incident to an undertaking of this sort. We can readily imagine many of those with which the conductors of the London Forceps had to contend. But we had hoped, that after having continued their paper one year, they had succeeded in securing for it a sufficiently extensive circulation to render its perpetuity certain. It had some able writers among its contributors, whose labors in this field of useful industry, we hope, will not cease, because the publication of their own valuable paper has been discontinued. If we cannot enjoy the fruits of it through the medium of a publication of their own, we should be happy of the opportunity of enriching the pages of the American Journal of Dental Science with them.—*Balt. Ed.*

